# Availability of Mobile Crisis Services in Mental Health Facilities

**DOI:** 10.1001/jamanetworkopen.2024.61321

**Published:** 2025-02-24

**Authors:** Andrew Anderson, Stas Spivak, Alene Kennedy Hendricks

**Affiliations:** 1 Bloomberg School of Public Health, Department of Health Policy and Management, Johns Hopkins University, Baltimore, Maryland; 2 Psychiatry Mobile Treatment, Psychiatry and Behavioral Sciences, Department of Mental Health, School of Medicine, Bloomberg School of Public Health, Johns Hopkins University, Baltimore, Maryland

## Abstract

**Importance:**

Rates of suicide ideation, severe depression, and acute psychosis have surged, paralleling an increase in emergency department visits for psychiatric emergencies. Mobile crisis services can play an important role in delivering timely, community-based interventions.

**Objective:**

To assess the national availability of mobile crisis services in mental health treatment facilities and to identify key facility, geographic, and state-level factors associated with the availability of these services.

**Design, Setting, and Participants:**

This cross-sectional study analyzed data from the 2022 National Substance Use and Mental Health Services Survey, which included 9036 mental health treatment facilities in the US. Facility zip codes were linked to the Agency for Healthcare Research and Quality’s Social Determinants of Health database to assess area-level factors. The study population included facilities that reported whether they offered mobile crisis services. Data were analyzed from August to September 2024.

**Main Outcomes and Measures:**

The primary outcome was the availability of mobile crisis services at mental health treatment facilities, as reported by facility directors. Key independent variables included facility characteristics, local area factors, and state Medicaid policies. Logistic regression models were used to examine the associations between these factors and the availability of mobile crisis services.

**Results:**

Of 9036 facilities, 1882 (20.8%) reported offering mobile crisis services. Facilities providing integrated dual diagnosis services (marginal effect, 3.44 [95% CI, 1.41-5.53] percentage points), suicide prevention services (marginal effect, 6.74 [95% CI, 4.29-9.20] percentage points), and assertive community treatment (marginal effect, 11.26 [95% CI, 9.02-13.51] percentage points) were significantly more likely to offer mobile health crisis services. Facilities in areas with lower percentages of residents with only a high school education were also more likely to offer mobile crisis services (marginal effect, 1.33 [95% CI, 0.15-2.51] percentage points).

**Conclusions and Relevance:**

In this cross-sectional study of US mental health facilities, facilities with integrated services and located in areas with certain geographic characteristics were more likely to offer mobile crisis services. Addressing gaps in service availability is essential to improving access to crisis care.

## Key Points

**Question:**

What facility, local area, and state policy characteristics are associated with the availability of mobile crisis services at mental health treatment facilities in the US?

**Findings:**

In this cross-sectional study of 9036 US facilities, 20.8% reported offering mobile crisis services. Facilities with integrated dual diagnosis services, suicide prevention, and assertive community treatment were more likely to provide mobile crisis services.

**Meaning:**

These findings suggest that facility-level characteristics and the local and state contexts in which they operate are associated with the availability of mobile crisis services, highlighting opportunities to expand access through targeted resource allocation and service integration.

## Teaser

This cross-sectional study describes the national landscape of mental health treatment facilities that offer mobile crisis services and examines key facility and local area–level factors associated with the availability of these services.

## Introduction

Rates of suicide ideation, severe depression, acute psychosis, and other risk factors for psychiatric emergencies have surged over the past decade, paralleling a 3-fold increase in emergency department visits related to mental health (MH).^1,2,3,4^ Increases span all age groups, with the most pronounced increase among adolescents.^5,6^ Psychiatric emergencies are acute disruptions in thoughts, behavior, mood, or social interactions that require immediate intervention to prevent imminent harm to the individual or others.^7^ Since the 1970s, mobile crisis services have been instrumental in responding to psychiatric emergencies. The launch of the 988 Suicide & Crisis Lifeline in July 2022^8,9^ underscores the importance of strengthening the mobile crisis infrastructure.

Mobile crisis services are an important component of the MH care continuum, providing “anyone, anywhere, at any time” access to assessment, treatment, and recovery programs.^10^ These services are part of a broader array of crisis interventions with varying definitions of *mobile*, generally encompassing services provided outside a facility or services that coordinate care offsite, such as mobile care coordination, crisis transportation, mobile addiction outreach, and others.^10^ One common model for delivering mobile crisis services is the mobile crisis team (MCT), which consists of health professionals deployed directly to individuals experiencing a crisis. MCTs practice trauma-informed care, de-escalation, and harm-reduction strategies.^11^ Research suggests that MCTs can effectively divert individuals from unnecessary emergency department visits,^12^ reduce hospital admissions,^13,14,15^ and prevent criminal justice involvement.^16^

By offering onsite assessments and connecting individuals with MH crises to longitudinal care services, mobile crisis services ensure a public health–focused approach to crisis intervention. The American Rescue Plan Act (ARPA) of 2021 increased federal funding to expand access to mobile crisis services, offering an 85% federal match for participating states’ expenditures on MCTs, effective April 2022.^17,18^ Additionally, the Centers Medicare & Medicaid Services (CMS) awarded $15 million in planning grants to state Medicaid agencies to develop state plan amendments, Section 1115 demonstration applications, and Section 1915(b) and 1915(c) waiver requests to further enhance community-based mobile crisis services.^19^

Despite the increased federal support through ARPA, there remains a significant gap in understanding where mobile crisis services sit within the broader MH care infrastructure. Understanding the types of MH facilities offering mobile crisis services is important because these facilities may also provide other specialized behavioral health services relevant to individuals in crisis. This could plausibly allow for a more coordinated response to MH crises, while also supporting a smoother transition to ongoing treatment and support.^20^ This study sought to assess the national landscape of MH treatment facilities that offer mobile crisis services and to identify key facility and local area–level factors that are associated with the availability of these services.

## Methods

This cross-sectional study was exempt from institutional review board approval and informed consent because it used publicly available, deidentified data on organizations, not individuals. The study adhered to the Strengthening the Reporting of Observational Studies in Epidemiology (STROBE) reporting guideline.

### Data

We used data from the 2022 National Substance Use and Mental Health Services Survey (N-SUMHSS) to compare the characteristics of MH treatment facilities with and without mobile crisis services in the US. The N-SUMHSS is a comprehensive annual survey conducted by the Substance Abuse and Mental Health Services Administration. The survey includes federal, state, and local government facilities as well as private facilities that provide MH treatment services. In 2022, this survey included data on 9586 MH treatment facilities (82% of 11 647 facilities), 14 854 substance use disorder (SUD) treatment facilities (86% of 17 353 facilities), and 3280 facilities providing both SUD and MH services (83% of 3935 facilities).

### Data Sources and Study Population

The 2022 N-SUMHSS was fielded from March 31 through December 4, 2022. The overall response rate was 88%. The survey sampling frame excluded Department of Defense military MH treatment facilities, individual private practitioners or small-group practices not licensed as SUD and/or MH clinics or centers, and jails. Residential facilities not primarily focused on specialty MH treatment were also excluded. Using facility zip codes from the 2023 National Directory of MH Treatment Facilities, which complements the 2022 N-SUMHSS survey, we also incorporated zip code–level information from the Agency for Healthcare Research and Quality’s database on Social Determinants of Health (SDOH) for the years 2011 to 2020. The SDOH database includes variables across 5 key domains: social context, economic context, education, physical infrastructure, and health care context. This study included data on facilities that provided MH services and had complete data on the availability of mobile crisis services, resulting in a final analytic sample of 9036 facilities.

### Study Outcome

The primary outcome was the availability of mobile crisis services at MH treatment facilities. Facility directors were asked “Does this facility offer mobile/off-site psychiatric crisis services?” and responded yes or no.

### Independent Variables

The study’s key independent variables include facility characteristics, zip code–level characteristics, and state-level policies. Facility characteristics were binary variables (0 or 1) indicating whether specific services are offered, including cognitive behavioral therapy, integrated MH and SUD treatment, suicide prevention, integrated MH treatment with primary care,^21^ case management, assertive community treatment, and psychiatric emergency walk-in. Housing services, vocational rehabilitation, and education services were included in the model as they address health-related social needs that influence MH outcomes. Housing services support individuals in securing stable living environments,^22^ while vocational rehabilitation helps individuals regain employment,^23^ fostering long-term recovery.^24^ Education services (eg, basic literacy, advanced education, and special education) promote self-sufficiency and health literacy by providing pathways to improved cognitive and functional abilities, enhancing overall stability and well-being.^25,26^ Zip code–level factors included the demographic characteristics where the facility was located, including: population density (measured as the number of people per square mile); median age; percentages of population that are uninsured, Medicaid enrolled, receive disability assistance, use public transit, live in a household without a personal vehicle; and have only a high school diploma (aged ≥25 years). We also included the mean distance (in miles) from the center of the zip code where the facility is located to the nearest hospital SUD inpatient care, as well as the nearest health clinic (eg, Federally Qualified Health Centers, Rural Health Centers). These measures were drawn from the AHRQ SDOH data sources, which pull from multiple publicly available data sources.

In terms of state-level policy factors, we identified states with ARPA 85% Medicaid match approval based on data indicating whether a state had received approval for a state plan amendment as of June 2022.^17^ Additionally, we identified states that received mobile crisis–related planning grants from CMS. Finally, we identified states that had expanded Medicaid as of 2022, recognizing that Medicaid expansion is a critical factor in broadening access to MH services, including mobile crisis interventions.^27^ In addition, we calculated state-level ratios of MH treatment facilities offering mobile crisis services to the total number of MH facilities in each state.

### Statistical Analysis

We conducted a bivariate analysis comparing all facility, zip code–level, and state policy characteristics among MH facilities with and without mobile crisis services. Pearson χ^2^ tests were used to compare categorical variables, and independent *t* tests were used to assess differences in means for continuous variables between facilities with and without mobile crisis services.

Multivariable logistic regression models were used to identify facility, zip code–level, and state policy characteristics associated with the availability of mobile crisis services. To account for the fact that multiple facilities can be located in the same zip code, SEs were clustered at the zip code level to adjust for potential correlation among facilities within the same geographic area. Postestimation marginal effects were calculated for all logistic regression models to translate the model coefficients into percentage point changes in the probabilities of offering mobile crisis services associated with a one-unit change in each independent variable.

All statistical analyses were conducted using Stata software version 17.0 (StataCorp). Statistical significance was set at a 2-sided *P* < .05. Data were analyzed from August to September 2024.

## Results

**Figure 1.**
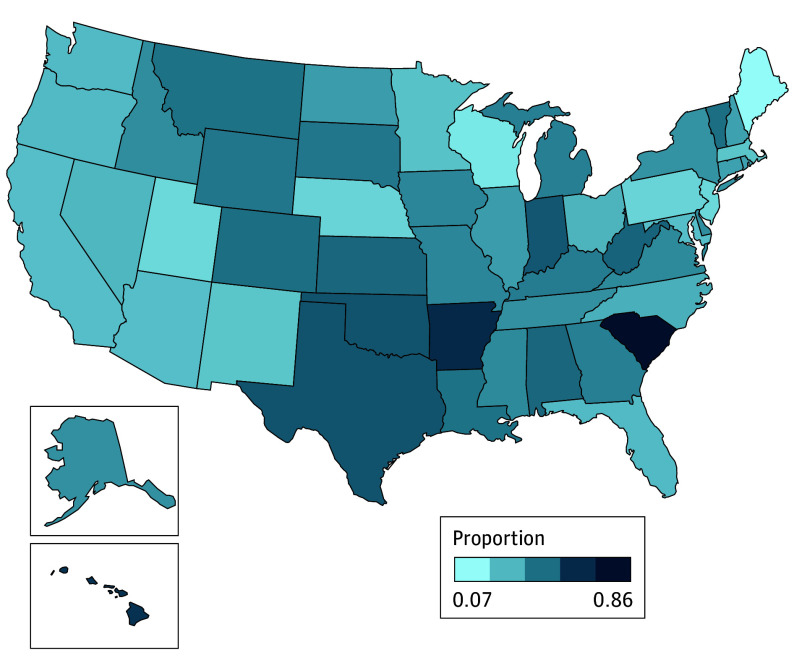
State-Specific Proportions of Mental Health Treatment Facilities Offering Mobile Crisis Services, 2022 The map illustrates the state-specific proportions of surveyed mental health treatment facilities that reported offering mobile crisis services.

**Figure 2.**
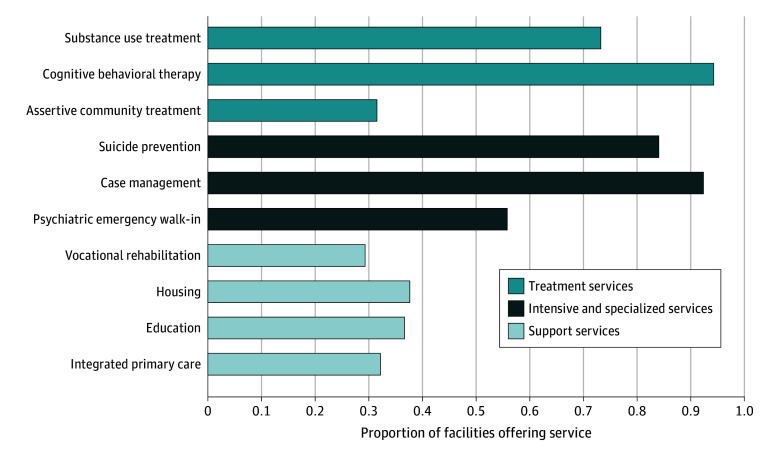
Service Offerings Among Facilities Providing Mobile Crisis Services, 2022 (n = 1879) Treatment services include interventions addressing mental health and substance use disorders. Intensive and specialized services provide targeted care for severe or acute conditions. Support services offer resources to help individuals maintain long-term stability and recovery.

**Figure 3.**
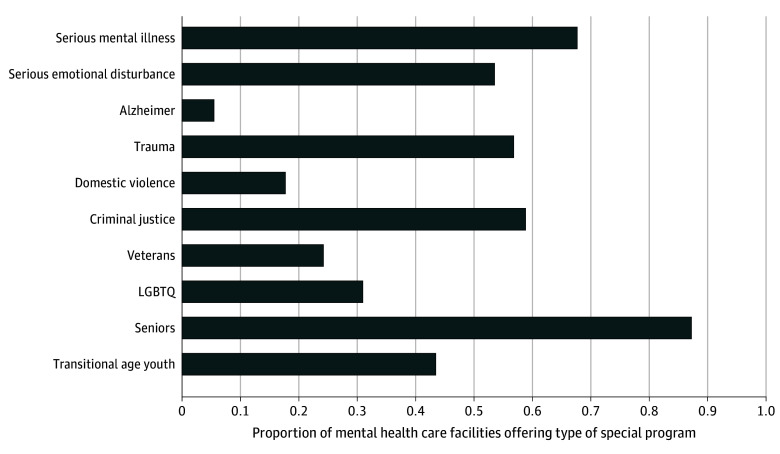
Population-Focused Programs Offered by Facilities Providing Mobile Crisis Services (n = 1879) Population-focused programs are specialized services designed to meet the unique needs of specific client groups, including individuals with serious mental illness, trauma survivors, veterans, and other identified populations. LGBTQ indicates lesbian, gay, bisexual, transgender, and/or queer.

We found 1882 facilities of 9586 total MH facilities (or 20% overall) that reported having mobile crisis services in 2022 (Table 1). There was significant variation across states, with proportions ranging from as low as 0.07 in some states to as high as 0.86 in others (Figure 1). For instance, South Carolina had the highest proportion of facilities offering these services, at 0.86, followed by Arkansas, at 0.71, and Texas, at 0.56. In contrast, states like Maine (0.07), Wisconsin (0.14), and Utah (0.18) had much lower proportions, reflecting notable differences in crisis service availability within the MH infrastructure. Most facilities offering mobile crisis service offered substance use treatment (1433 facilities [76.1%]) and cognitive behavioral therapy (1774 facilities [94.3%]), while fewer provided assertive community treatment (593 facilities [31.6%]) or vocational rehabilitation services (552 facilities [29.3%]), highlighting variability in the availability of integrated services across facilities (Figure 2). In addition, most facilities offering mobile crisis services offered population-focused programs for individuals with serious mental illness and serious emotional disturbance (Figure 3). Other common programs focused on trauma, criminal justice involvement, and transitional-age youth, while programs for other populations, such as individuals with Alzheimer disease or domestic violence survivors, were less frequently offered.

**Table 1.** Comparison of Facility and County Characteristics Between Facilities With and Without Mobile Crisis Services

Characteristic	Facilities, mean (SD)		*P* value^a^
	No mobile crisis services (n = 7154)	Mobile crisis services (n = 1882)	
Services offered, No. (%)			
Substance use treatment	4424 (61.8)	1433 (76.1)	<.001
Cognitive behavioral therapy	6485 (9.7)	1774 (94.3)	<.001
Assertive community treatment	841 (11.8)	593 (31.6)	<.001
Suicide prevention	4678 (65.4)	1582 (84.3)	<.001
Case management	5286 (73.9)	1738 (92.4)	<.001
Psychiatric emergency walk-in	1770 (24.7)	1051 (55.8)	<.001
Vocational rehabilitation	970 (13.6)	552 (29.3)	<.001
Housing assistance	1344 (18.8)	708 (37.6)	<.001
Education assistance	2738 (37.3)	690 (36.7)	.20
Integrated primary care	1864 (26.1)	605 (32.2)	<.001
Area demographic characteristics			
Population density, people per square mile	4089.70 (10 969.67)	2916.14 (9512.84)	<.001
Uninsured, %	8.11 (5.14)	9.04 (5.13)	<.001
Medicaid beneficiaries, %	19.15 (11.02)	2.58 (1.67)	<.001
Disabled residents, %	14.01 (5.20)	15.47 (5.39)	<.001
Age, median (IQR), y	38.7 (35.1-42.4)	39.2 (34.7-42.3)	.01
Residents without a vehicle, %	5.27 (2.60)	5.44 (2.65)	.01
Residents using public transit, %	5.28 (11.11)	3.90 (1.11)	<.001
Only high school education, %	27.50 (9.66)	3.11 (9.19)	<.001
Geographic characteristics			
Distance to alcohol treatment facilities, mile	9.60 (17.85)	15.65 (23.31)	<.001
Distance to clinics, mile	3.07 (7.99)	2.68 (5.23)	.046
ARPA SPA approved, No. (%)	580 (9.7)	124 (7.5)	.001
Non-ARPA coverage, No. (%)	2411 (40.1)	736 (44.3)	.001
State planning grants, No. (%)	3016 (50.2)	800 (48.2)	.001
Nonexpansion state, No. (%)	1245 (18.1)	412 (22.9)	<.001
Expansion state, No. (%)	5652 (82.0)	1386 (77.1)	<.001

Abbreviations: ARPA, American Rescue Plan Act; SPA, State Plan Amendment.

^a^ Statistical testing was performed using χ2 tests for categorical variables and *t* tests for continuous variables.

### Facility Characteristics

Facilities offering mobile crisis services, compared with facilities without mobile crisis services, were more likely to provide cognitive behavioral therapy (94.3% vs 9.7%; *P* < .001), integrated MH and SUD treatment (76.1% vs 61.8%; *P* < .001), and suicide prevention services (84.3% vs 65.4%; *P* < .001). Facilities with mobile crisis services were also more likely to offer case management services (92.4% vs 73.9%; *P* < .001), assertive community treatment (31.6% vs 11.8%; *P* < .001), and psychiatric emergency walk-in services (55.8% vs 24.7%; *P* < .001). Housing services (37.6% vs 18.8%, *P* < .001) and vocational rehabilitation services (29.3% vs 13.6%, *P* < .001) were also more prevalent in facilities with mobile crisis services than facilities without.

### Demographic and Geographic Characteristics

Facilities with mobile crisis services, compared with facilities without, were located in areas with lower population density (mean [SD], 2916.14 [9512.84] vs 4089.70 [10 969.67] people per 1 square mile; *P* < .001) and had higher percentages of uninsured patients (mean [SD], 9.04% [5.13%] vs 8.11% [5.14%]; *P* < .001), Medicaid beneficiaries (mean [SD], 2.58% [1.67%] vs 19.15 [11.02%]; *P* < .001), and residents with disabilities (mean [SD], 15.47% [5.39%] vs 14.01% [5.20%]; *P* < .001). Additionally, areas with mobile crises services had a lower percentage of individuals with only a high school diploma (mean [SD], 3.11% [9.19%] vs 27.50% [9.66%]; *P* < .001) and greater distances to alcohol treatment facilities (mean [SD], 15.65 [23.31] vs 9.60 [17.85] miles; *P* < .001). The population was older in areas with mobile crisis services (median [IQR] age, 39.2 [35.1-42.4] vs 38.7 [34.7-42.3] years; *P* = .01), and the distance to clinics was shorter (mean [SD], 2.68 [5.23] vs 3.07 [7.99] miles; *P* = .046).

### State-Level Characteristics

A lower proportion of facilities offering mobile crisis services were in states with ARPA state plan amendment approval (7.5% vs. 9.7%; *P* = .001). Conversely, a higher proportion were in states with non-ARPA coverage (44.3% vs. 40.1%; *P* = .001). Facilities in states with planning grants were similarly distributed, with 48.2% of facilities offering mobile crisis services compared with 50.2% without (*P* = .001). Facilities in nonexpansion states were more likely to offer mobile crisis services compared with those in expansion states (22.9% vs 18.1%; *P* < .001).

### Factors Associated With Mobile Crisis Service Availability

Facilities providing integrated dual diagnosis services (marginal effect, 3.44 [95% CI, 1.41 to 5.53] percentage points; *P* < .001), suicide prevention services (marginal effect, 6.74 [95% CI, 4.29 to 9.20] percentage points; *P* < .001), and assertive community treatment (marginal effect, 11.26 [95% CI, 9.02 to 13.51] percentage points; *P* < .001) were significantly more likely to offer mobile crisis services (Table 2). Additionally, case management services (marginal effect, 12.80 [95% CI, 9.93 to 15.67] percentage points; *P* < .001), psychiatric emergency walk-in services (marginal effect, 12.04 [95% CI, 10.14 to 13.94] percentage points; *P* < .001), vocational rehabilitation services (marginal effect, 5.55 [95% CI, 2.93 to 8.17] percentage points; *P* < .001), and housing services (marginal effect, 6.39 [95% CI, 4.15 to 8.63] percentage points; *P* < .001) were positively associated with mobile crisis service availability. Conversely, facilities offering integrated primary care (marginal effect, −3.80 [95% CI, −5.96 to −1.63] percentage points; *P* < .001) and education services (marginal effect, −6.35 [95% CI, −8.32 to −4.37] percentage points; *P* < .001) were less likely to provide mobile crisis services.

**Table 2.** Changes in the Probability of Mobile Crisis Availability Associated With Facility, Demographic, and Geographic Characteristics on Mobile Crisis Service Availability

Characteristics	Marginal effect (95% CI), percentage points	*P* value
Services offered		
Cognitive behavioral therapy	−1.35 (−4.95 to 2.25)	.68
Integrated dual diagnosis	3.44 (1.41 to 5.53)	<.001
Suicide prevention services	6.74 (4.29 to 9.20)	<.001
Integrated primary care	−3.80 (−5.96 to −1.63)	.01
Case management	12.8 (9.93 to 15.67)	<.001
Assertive community treatment	11.26 (9.02 to 13.51)	<.001
Psychiatric emergency walk-in	12.04 (10.14 to 13.94)	<.001
Vocational rehabilitation	5.55 (2.93 to 8.17)	.004
Housing assistance	6.39 (4.15 to 8.63)	<.001
Education assistance	−6.35 (−8.32 to −4.37)	<.001
Geographic characteristics		
Population density, per 1000 people per square mile	−0.00117 (−0.00147 to 0.00123)	.87
Uninsured residents, per 10 percentage points	−0.12 (−1.90 to 1.65)	.89
Medicaid beneficiaries, per 10 percentage points	−0.24 (−1.41 to 0.94)	.69
Disabled residents, per 10 percentage points	2.08 (−0.23 to 4.39)	.08
Median age, per 1 y	−1.6 (−3.21 to 0.02)	.05
Transportation access challenges	−1.42 (−4.46 to 1.61)	.36
Public transit use, per 10 percentage points population	0.28 (−0.93 to 1.49)	.65
Only high school education, per 10 percentage points	1.33 (0.15 to 2.51)	.03
Distance to alcohol treatment facility, per 1 mile	1.88 (1.24 to 2.53)	<.001
Distance to clinic, per 1 mile	−1.44 (−3.56 to 0.68)	.18
State-level policy characteristics		
Medicaid expansion	0.26 (−2.02 to 2.54)	.82
APRA match	1.84 (−0.93 to 4.61)	.19
State planning grants	2.09 (−0.57 to 4.74)	.12

Abbreviation: ARPA, American Rescue Plan Act.

Among demographic factors, the percentage of residents with only a high school education (marginal effect, 1.33 [95% CI, 0.15 to 2.51] percentage points; *P* = .03) was positively associated with mobile crisis service availability. Greater distance to alcohol treatment facilities was associated with an increased likelihood of offering mobile crisis services (marginal effect, 1.88 [95% CI, 1.24 to 2.53] percentage points per mile; *P* < .001). State-level policies, including ARPA State Plan Amendment approval, were not significantly associated with the availability of mobile crisis services after adjusting for facility, zip code-level, and other state policy factors (Table 2).

## Discussion

In this cross-sectional study using 2022 national data on MH care facilities in the US, we found that more than 20% percent of facilities reported offering mobile crisis services. Facilities offering other relevant services, such as dual diagnosis treatment, suicide prevention, and case management, were more likely to provide mobile crisis services. Mobile crisis services within MH treatment facilities can play a role in providing accessible and effective crisis intervention. Service integration with mobile crisis services could improve care by allowing individuals in crisis to receive immediate, coordinated treatment without navigating multiple systems or referrals.^28^ Similarly, facilities with assertive community treatment and psychiatric emergency walk-in services were also more likely to offer mobile crisis services, suggesting that facilities that have other types of community-based services (eg, assertive community treatment) or crisis response services (ie, emergency walk-in services) are also more likely to offer mobile crisis services.

In a 2022 national survey, 44 states reported supporting MCTs, one model of mobile crisis service. However, only 24 states have statewide MCT coverage and even fewer (20 states) have teams available 24 hours a day, 7 days a week. Challenges persist, particularly in rural areas, due to staffing and operational difficulties. Despite these efforts, only one-third of states monitor outcomes, with many MCTs not yet fully integrated across the US.^29^ This limited coverage and inconsistent monitoring stress the need for a deeper understanding of the factors influencing mobile crisis service availability.^30^ Notably, this survey focused exclusively on MCTs, whereas our analysis encompasses a broader scope of mobile crisis services.

We found that the states in the southern US, particularly in South Carolina, Arkansas, and Texas, had the highest unadjusted proportion of MH facilities offering mobile crisis services. However, service availability likely depends, in part, on differences in population composition, MH care infrastructure, and state policies.^31^ For instance, the national median age-adjusted suicide rate in 2022 was 16.0 deaths per 100 000. The rates were 15.4 deaths, 18.0 deaths, and 14.4 deaths per 100 000 in South Carolina, Arkansas, and Texas, respectively.^32^ South Carolina implemented a community crisis response and intervention program that provides statewide crisis intervention services 24 hours a day, 7 days a week, integrating MCTs into the broader MH care system in all 46 counties.^33^

The lower proportion of facilities with mobile crisis services in states with ARPA state amendment approval for the 85% match, compared with nonexpansion states, may reflect differences in need for federal funding and the timing of policy implementation. Given that ARPA was enacted in 2021 and the data for this study were collected in 2022, these states may have a greater need for funds to build up their mobile crisis infrastructure. The lower proportions may reflect the early stages of policy implementation. Another study found counties in states with legislation funding the 988 Suicide & Crisis Lifeline were more likely to have MCTs, while states with 115 waivers restricting Medicaid benefits were less likely to have MCTs.^34^ This variation suggests that the approach to implementing mobile crisis services can differ significantly based on state policies, available funding, and the time for operationalizing these changes.

We found that facilities that offered mobile crisis services were also more likely to provide a range of other important services, including cognitive behavioral therapy, integrated MH and SUD treatment, and suicide prevention services. These facilities also more commonly offered supportive services, like case management and assertive community treatment. These service offerings can enhance the effectiveness of crisis intervention by addressing both the immediate crisis and underlying MH problems, as well as facilitating continuity of care. By integrating these services, facilities ensure a more holistic approach to patient care and recovery.^35^

Facility-level characteristics, rather than geographic or demographic factors, were associated with the availability of mobile crisis services. Although these services were located in zip codes with higher needs (eg, higher rates of disability, uninsured populations, or greater distances to SUD treatment facilities), this does not necessarily mean that the needs are being fully met relative to the demand.

### Limitations

This study has some limitations. Our study was cross-sectional, and we were unable to observe changes over time in mobile crisis service availability. However, this design still provides a valuable snapshot of the current landscape, capturing key characteristics of service availability during the transition to the 988 Suicide & Crisis Lifeline.^36^ The N-SUMHSS provides the most recent nationally representative data on MH facilities; however, it does not include all facilities, such as certain hospital systems or standalone MCTs. These exclusions may result in underestimations of mobile crisis service availability. Despite these limitations, the dataset remains valuable for assessing trends and facility-level characteristics. While this study provides insights into facility-level availability of mobile crisis services, it does not directly reflect coverage. A single facility with regional coverage may serve more residents than multiple localized facilities. Our analysis was conducted at the zip code level, recognizing that multiple facilities may exist within a single geographic area. Facilities within the same zip code or nearby areas may share unmeasured characteristics, potentially leading to spatial dependencies. Although our inclusion of area-level covariates, such as percentages of disabled residents and residents with only a high school education, helps address these dependencies, we acknowledge that residual spatial autocorrelation may remain. Moreover, the AHRQ SDOH database used in our analysis covers the period from 2011 to 2020, while the facilities were surveyed in 2022. Although there is a temporal mismatch, area-level characteristics are generally stable over short periods and using the 2011 to 2020 data reduces the risk of capturing random year-to-year fluctuations, providing a more consistent and reliable view of community characteristics.

## Conclusions

This cross-sectional study examined the facility, local area, and state policy characteristics associated with the availability of mobile crisis services in MH health treatment facilities across the US. These findings suggest that both the characteristics of facilities as well as their local area characteristics were associated with availability of mobile crisis services. MH treatment facilities, as key providers of behavioral health services, could serve as a hub for mobile crisis services where possible to enhance continuity of care, foster integration, and minimize disruptive hand-offs to external health care practitioners. Although more than 20% of MH facilities reported offering these services, it remains uncertain whether they adequately meet the demand for care. Mobile crisis services were more frequently available in areas with higher proportions of Medicaid beneficiaries, uninsured individuals, and people with disabilities, populations that typically encounter significant barriers to accessing care. Additionally, facilities offering mobile crisis services often provided other services important for the treatment and recovery of MH and SUD. Future research should investigate the utilization of mobile crisis services, their impact on care continuity, and their effects on health outcomes.

## Notes

Supplement.
Data Sharing Statement
