# Supplementary material for: Availability of Mobile Crisis Services in Mental Health Facilities
Source: JAMA Netw Open. 2025 Feb 24;8(2):e2461321. doi: 10.1001/jamanetworkopen.2024.61321 (PMC11851237; doi:10.1001/jamanetworkopen.2024.61321)
Supplement: Supplement. — Data Sharing Statement [file jamanetwopen-e2461321-s001.pdf]

Data Sharing Statement

Anderson. Availability of Mobile Crisis Services in Mental Health Facilities. *JAMA Netw Open*. Published February 24, 2025. doi:10.1001/jamanetworkopen.2024.61321

Data
Data available: No

Additional Information
Explanation for why data not available: The data are publicly accesible.
